# Lytic Infection with Murine Gammaherpesvirus 68 Activates Host and Viral RNA Polymerase III Promoters and Enhances Noncoding RNA Expression

**DOI:** 10.1128/JVI.00079-21

**Published:** 2021-06-24

**Authors:** Ashley N. Knox, Alice Mueller, Eva M. Medina, Eric T. Clambey, Linda F. van Dyk

**Affiliations:** a Department of Immunology and Microbiology, University of Colorado Anschutz Medical Campus, Aurora, Colorado, USA; b Department of Molecular, Cellular, and Developmental Biology, University of Colorado, Boulder, Colorado, USA; c Department of Anesthesiology, University of Colorado Anschutz Medical Campus, Aurora, Colorado, USA; Northwestern University

**Keywords:** gammaherpesvirus, noncoding RNA, RNA polymerase III, transcriptional regulation

## Abstract

RNA polymerase III (pol III) transcribes multiple noncoding RNAs (ncRNAs) that are essential for cellular function. Pol III-dependent transcription is also engaged during certain viral infections, including those of the gammaherpesviruses (γHVs), where pol III-dependent viral ncRNAs promote pathogenesis. Additionally, several host ncRNAs are upregulated during γHV infection and play integral roles in pathogenesis by facilitating viral establishment and gene expression. Here, we sought to investigate how pol III promoters and transcripts are regulated during gammaherpesvirus infection using the murine gammaherpesvirus 68 (γHV68) system. To compare the transcription of host and viral pol III-dependent ncRNAs, we analyzed a series of pol III promoters for host and viral ncRNAs using a luciferase reporter optimized to measure pol III activity. We measured promoter activity from the reporter gene at the translation level via luciferase activity and at the transcription level via reverse transcription-quantitative PCR (RT-qPCR). We further measured endogenous ncRNA expression at single-cell resolution by flow cytometry. These studies demonstrated that lytic infection with γHV68 increased the transcription from multiple host and viral pol III promoters and further identified the ability of accessory sequences to influence both baseline and inducible promoter activity after infection. RNA flow cytometry revealed the induction of endogenous pol III-derived ncRNAs that tightly correlated with viral gene expression. These studies highlight how lytic gammaherpesvirus infection alters the transcriptional landscape of host cells to increase pol III-derived RNAs, a process that may further modify cellular function and enhance viral gene expression and pathogenesis.

**IMPORTANCE** Gammaherpesviruses are a prime example of how viruses can alter the host transcriptional landscape to establish infection. Despite major insights into how these viruses modify RNA polymerase II-dependent generation of messenger RNAs, how these viruses influence the activity of host RNA polymerase III remains much less clear. Small noncoding RNAs produced by RNA polymerase III are increasingly recognized to play critical regulatory roles in cell biology and virus infection. Studies of RNA polymerase III-dependent transcription are complicated by multiple promoter types and diverse RNAs with variable stability and processing requirements. Here, we characterized a reporter system to directly study RNA polymerase III-dependent responses during gammaherpesvirus infection and utilized single-cell flow cytometry-based methods to reveal that gammaherpesvirus lytic replication broadly induces pol III activity to enhance host and viral noncoding RNA expression within the infected cell.

## INTRODUCTION

Gammaherpesviruses (γHVs) are large, double-stranded DNA viruses that establish a lifelong infection in their hosts, with long-term latency in lymphocytes (1, 2). The γHVs include the human pathogens Epstein-Barr virus (EBV), Kaposi’s sarcoma-associated herpesvirus (KSHV or HHV-8), and murine gammaherpesvirus 68 (γHV68 or MHV-68; ICTV nomenclature *Murid herpesvirus 4*, MuHV-4) (3). These viruses establish a primary lytic infection in their host that is followed by a prolonged quiescent infection termed latency. Latency is maintained in healthy individuals by a homeostatic relationship between the virus and the host immune response; if this balance is disrupted (e.g., by immunosuppression), γHVs can reactivate from latency and actively replicate. Disruption between the balance of γHV infection and host immune control is associated with multiple γHV pathologies, including a range of malignancies (4).

The γHVs contain several types of noncoding RNAs (ncRNAs), including nuclear ncRNAs and functional microRNAs (miRNAs); these diverse RNAs include ncRNAs transcribed by RNA polymerase II (e.g., the KSHV PAN RNA and the KSHV and EBV miRNAs) or by RNA pol III (e.g., the EBV-encoded small RNAs [EBERs] and the γHV68 tRNA-miRNA-encoded RNAs [TMERs]) (5–13). Viral ncRNAs are considered to have important host-modulatory functions, interacting with host proteins and regulating host and viral gene expression. For example, the EBV EBERs are expressed during latency and were discovered through their interaction with the host lupus-associated antigen (La) protein, which putatively mediates EBER interaction with TLR3 (14–17). The EBERs have further been shown to interact with several host proteins, including ribosomal protein L22, protein kinase R (PKR), and retinoic acid-inducible gene I (RIG-I) (18). These interactions can trigger sustained host innate immune responses that are implicated in the development of EBV-associated malignancies (16, 19–21). γHV68, a highly tractable small-animal model of γHV infection, also encodes several pol III-transcribed ncRNAs, known as the tRNA-miRNA-encoded RNAs (TMERs) (22, 23). The TMERs are dispensable for lytic replication and establishment of latency; however, these transcripts are required for pathogenesis during acute infection of an immunocompromised host (7, 24, 25). The TMERs contain bifunctional elements with a tRNA-like structure at the 5′ end and hairpins that are processed into biologically active miRNAs (7) capable of targeting a number of RNAs for posttranscriptional regulation (26). Our laboratory has previously shown that the tRNA-like structure is sufficient to rescue pathogenesis of a TMER-deficient viral recombinant, suggesting that, like the EBERs, the TMERs contribute to pathogenesis through their interactions with host proteins (25). Although TMER-host protein interactions have yet to be fully explored, it is notable that several characteristics of the EBERs, such as a 5′-triphosphate and 3′-polyU, are imparted by RNA polymerase III (pol III) transcription (27). These motifs can be recognized by host RNA-binding proteins, such as RIG-I or La, to trigger an innate immune response (16, 20, 27, 28).

Pol III is often considered to perform housekeeping functions, as it transcribes host genes required for cell growth and maintenance (e.g., U6 snRNA, tRNAs, and 5S rRNA) (29). Despite this, it is clear that the γHVs can usurp pol III-dependent transcription mechanisms for their own purposes. Latent EBV infection has been shown to upregulate components of pol III and, ultimately, increase the expression of host pol III transcripts, particularly vault RNAs, that allow increased establishment of viral infection and gene expression (30–32). Similarly, γHV68 infection drives upregulation of host pol III-dependent short interspersed nuclear element (SINE) RNAs, which, in turn, mediate increased viral gene expression (33–35). Additionally, our laboratory has reported that reactivation of a latently infected γHV68 cell line results in a rare subset of the population that demonstrates increased viral transcription and translation, including increased expression of TMERs (36). Notably, dysregulation of pol III is a common feature of many cancer cells, implicating γHV infection-driven alteration of pol III activity as one contributor to γHV-associated malignancies (37). Therefore, understanding how γHV infection alters pol III activity is integral to elucidating mechanisms of γHV pathogenesis.

The analysis of pol III activity during γHV infection has been complicated by the nature of pol III-derived ncRNAs. These transcripts are often short and structured, creating complications in probe specificity to quantitatively analyze promoter activity/gene expression by conventional means (e.g., Northern blotting and reverse transcription-quantitative PCR [RT-qPCR]). Probe specificity is also challenging for classes of RNAs with highly conserved promoter features, such as the γHV68 TMERs or the human tRNAs. However, promoter analysis of the TMERs could reveal rules of transcription that apply to other conserved and potentially coregulated ncRNAs, such as the human tRNAs. Additionally, many pol III-derived ncRNAs may be scarce or abundant, so changes in expression can be obscured. Therefore, highly sensitive readouts, such as the high dynamic range of luciferase assays or quantitative assays with single-cell resolution, offer potential improvement in measuring pol III-derived ncRNA expression. Single-cell RNA flow cytometry allows RNA detection with high specificity without the need for unique primers and probe and has the additional benefit of measuring RNA levels in individual cells to reveal fine fluctuations that may be obscured in bulk analyses.

The ideal comparison of promoters allows variation only in the promoter elements coupled to a common reporter gene and sensitive detection. Traditional analysis of RNA pol II promoter activity has benefitted significantly from the use of luciferase reporter systems, which provide the advantages of a readout that is high throughput, has a wide dynamic range for maximal quantitation, and is standardized across varied promoters and cellular conditions. RNA pol III does not produce coding RNAs in normal biology; however, several studies have reported the use of luciferase reporters to measure ncRNA derived from pol III or pol I (38, 39). Based on these studies, we developed a panel of luciferase reporters driven by pol III promoters to determine the efficacy of a reporter gene approach in analyzing ncRNA promoter activity during viral infection. As with analysis of RNA pol II reporters, caveats to the enzymatic readout of this system are that it is several steps downstream of RNA transcription, the efficacy of RNA translation may differ among specific RNAs, and infection may alter translation in a number of ways yet to be described. However, use of the facile enzymatic readout plus RT-qPCR quantitation of the reporter RNA allows us to directly compare these measures for highly sensitive quantitative analysis. As a complementary approach, we quantified ncRNAs expression at the single-cell level in the presence or absence of virus infection.

Due to the importance of γHV ncRNAs during infection and the unique transcriptional regulation afforded by RNA pol III, the overall objective of this study was to compare different RNA pol III promoters and their activity during virus infection using three different methods for sensitive and quantitative analysis. We found that γHV68 infection upregulates the activity of multiple viral and host pol III promoters, a process further associated with the induction of pol III-dependent targets. These studies indicate that lytic γHV infection can broadly enhance RNA pol III promoter activity to modify the ncRNA landscape of infected cells.

## RESULTS

**FIG 1:**
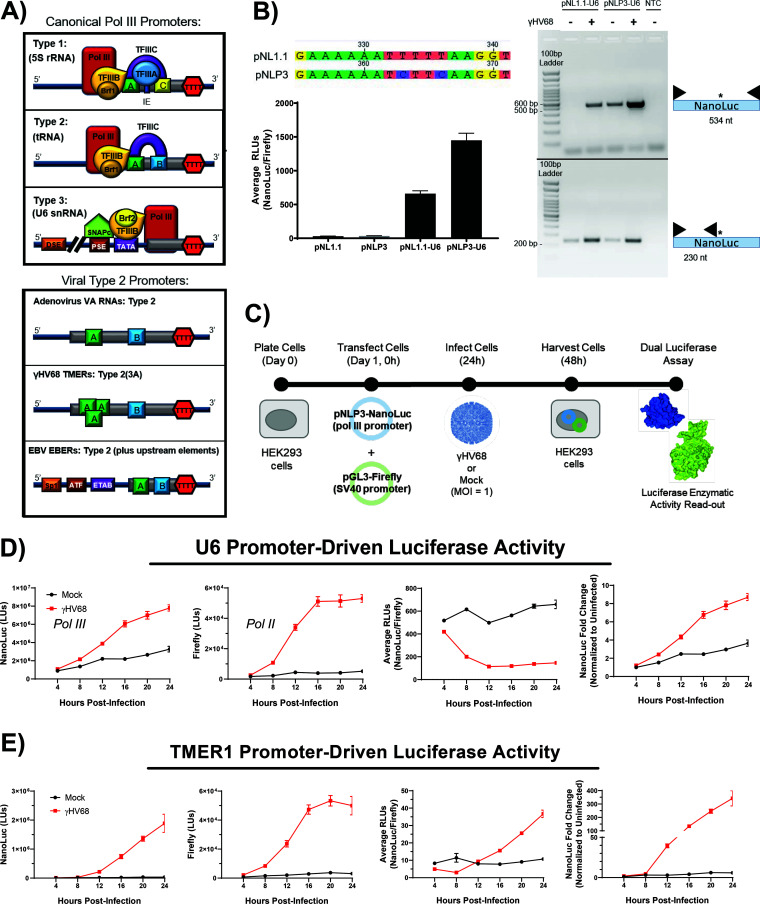
RNA polymerase III promoters contain distinct motifs and respond to viral infection. (A) Pol III transcribes RNA from gene-internal (type 1 and type 2) or gene-external (type 3) promoters. Each promoter type contains distinct motifs, which are, in turn, bound by specific transcription factors (TF) that recruit pol III to the promoter. Viral promoters can have canonical type 2 promoters, such as adenovirus; however, the γHV68 TMERs contain a triplicated, overlapping A box motif, and the EBV EBER promoters include upstream elements. Gray boxes indicate the gene, while TTTT marks the end of the gene where pol III transcription is terminated. Diagrams are not to scale. A, A box; B, B box; C, C box; IE, intermediate element; TTTT, pol III termination signal; TF, transcription factor; TATA, TATA box; PSE, proximal sequence element; DSE, distal sequence elements; ETAB, EBER TATA-like box; ATF, activating transcription factor binding sequence; Sp1 , Sp1-like binding sequence. (B) Optimization of the NanoLuc reporter vector. Mutations were introduced into the pNL1.1 vector to remove the pol III termination signal (TTTT) from the NanoLuc coding region, resulting in the pNLP3 vector. (Left) The human U6 promoter was cloned into each of these reporters, and dual-luciferase assays were performed to compare luciferase output. Data shown are representative of two independent experiments with biological triplicates. Additionally, RNA was isolated from cells transfected with these two constructs. (Right) Cells were mock or WT γHV68 infected for 16 h, and then cellular RNA was used as a template for primers targeting the entire NanoLuc gene (top gel, 534 nt) or targeting just the NanoLuc sequence upstream of the termination sequence (bottom gel, 234 nt) (with primers as indicated). Data shown are from one experiment with biological triplicates. NTC, nontemplate control; black triangles, primers; *, location of termination sequence in pNL1.1. (C) Experimental design. HEK 293 cells were transfected with a control Firefly reporter (pGL3) and a pNLP3 reporter expressed from a pol III promoter of interest. Twenty-four hours posttransfection, cells were infected with wild-type (WT) γHV68 at an MOI of 1 or mock treated. Cell lysates were collected 24 h posttransfection for the dual-luciferase assay. Promoter activity was measured for the U6 (D) and TMER1 (E) promoters using a dual-luciferase assay. Cells were harvested every 4 h postinfection up to 24 h. Each time point was repeated for biological triplicates. Raw values for the Firefly and NanoLuc activity for each promoter are shown as luminescence units (LUs). Promoter activity was analyzed as average relative luminescence units (RLUs; NanoLuc LUs/Firefly LUs) and as the fold change in NanoLuc luminescence units compared to the 4-h uninfected samples (NanoLuc fold change, Infected sample NanoLuc LU/mock sample NanoLuc LU). Error bars, standard errors of the means (SEM).

RNA polymerase III can transcribe RNA from a variety of gene-internal (type 1 and 2) and gene-external (type 3) promoters (Fig. 1A). These promoters contain distinct motifs that determine which transcription factors bind to the promoter to recruit pol III (40). To understand how γHV lytic replication influenced RNA pol III promoter activity while limiting the confounding factors of the individual RNA primary transcripts and modified or processed products, we sought to make use of a luciferase assay previously used to study pol III promoter activity (41) to study a series of viral and host pol III promoters. We selected the reporter plasmid pNL1.1 (Promega), because NanoLuc luciferase creates a brighter signal, and the protein is smaller than other luciferase proteins (NanoLuc, 19.1 kDa and 171 nucleotides [nt]; Renilla, 36.0 kDa and 312 nucleotides; Firefly, 60.6 kDa and 550 nucleotides), which is consistent with pol III processivity of small ncRNAs. Our analysis of the pNL1.1 sequence revealed a pol III termination signal within the luciferase coding gene (TTTT). Therefore, to examine the activity of pol III promoters without the potential for early termination, we introduced silent mutations into the NanoLuc reporter construct to remove the termination signal. This altered vector was named pNLP3 to reflect that it is a NanoLuc reporter optimized for pol III (Fig. 1B). The human U6 promoter was cloned into both the parental pNL1.1 and the modified pNLP3 to compare the effects of removing the pol III termination signal, with promoter activity measured 24 h postinfection. We found that removal of the termination signal increased the luciferase output, indicating that there was more readthrough of the full NanoLuc gene from pNLP3 (Fig. 1B, left). Furthermore, RT-PCR analysis of the NanoLuc transcript transcribed from the U6 promoter in either the pNL1.1 or pNLP3 vector revealed more full-length NanoLuc transcript from the pNLP3 vector (Fig. 1B, right). This indicates that the pNLP3 vector allows for optimal pol III transcription of the reporter gene. Therefore, we used the pNLP3 vector as the backbone for analysis of all other pol III promoters included in this study.

With an optimized pol III reporter construct, we assessed how different pol III promoters respond to γHV68 infection over time. We first compared the activity of the human U6 (type 3) and γHV68 TMER1 (type 2) promoters (Table 1). HEK 293 cells were transfected with pNLP3 vectors containing either the U6 or TMER1 promoter, cotransfected with a simian virus 40 (SV40) (pol II promoter)-driven Firefly luciferase vector and then infected with γHV68 (Fig. 1C). Cell lysates were collected every 4 h for 24 h postinfection to quantify promoter activity, as defined by NanoLuc luciferase activity. This analysis revealed that γHV68 infection resulted in a time-dependent increase in NanoLuc activity for both the U6 and TMER1 promoters (Fig. 1D and E, left) relative to mock-infected samples. γHV68 infection also resulted in a time-dependent increase in expression of the control, Firefly luciferase reporter (Fig. 1D and E, second panel from left). The results for dual-luciferase assays are typically reported as relative luminescence units (RLUs), where the reporter luminescence units (LUs) are normalized to the luminescence units of the control luciferase (i.e., NanoLuc LUs/Firefly LUs). However, since γHV68 infection simultaneously increased luminescence from both the NanoLuc reporter and the control Firefly reporter, this normalization implied decreased relative U6 promoter activity with infection when we actually observe an increase in NanoLuc activity (Fig. 1D). Clearly, the numerous changes incurred in cells during viral infection limits our ability to standardize pol III promoter activity relative to a pol II promoter control (i.e., SV40 promoter); therefore, all subsequent analyses report promoter activity as a fold change in NanoLuc luminescence comparing mock- and γHV68-infected samples. This allows us to directly compare the effect of infection on the reporter in related samples. We found that the U6 promoter drives high basal luciferase activity under mock conditions (Fig. 1D, left), with a further increase in raw and normalized U6-expressed NanoLuc LUs throughout infection (Fig. 1D). In contrast, the TMER1 promoter was characterized by extremely low basal luciferase activity (Fig. 1E, left) under mock conditions; however, this promoter was strongly induced by infection (Fig. 1E). These data suggest that the luciferase assay can be used for analysis of pol III promoters and show that γHV68 lytic infection increases the activity of multiple pol III promoter types, with a more robust induction of the type 2 promoter of TMER1 than the U6 promoter.

**TABLE 1:** Promoters analyzed in this study

Origin	Promoter	Promoter type^*a*^	Promoter insert length (nt)	Reference
Human	5S rRNA	Type 1	42	54
Human	tRNA-Tyr	Type 2	53	Gene ID 100189507
Human	Vault RNA1-1	Type 2	65	31, 55; GenBank accession no. AF045143.1
Human	U6 snRNA	Type 3	227	GenBank accession no. M14486.1
Adenovirus	VA1	Type 2	58	56
γHV68	TMER1 full	Type 2 (3A)	91	22
γHV68	TMER1 minimal	Type 2 (3A)	55	22
γHV68	TMER4 full	Type 2 (3A)	93	22
γHV68	TMER4 minimal	Type 2 (3A)	55	22
γHV68	TMER5 full	Type 2 (3A)	94	22
γHV68	TMER5 minimal	Type 2 (3A)	53	22
EBV	EBER1	Type 2 (+ upstream)	157	57–59
EBV	EBER1 minimal	Type 2 (no upstream)	70	57–59
EBV	EBER2	Type 2 (+ upstream)	156	57–59
EBV	EBER2 minimal	Type 2 (no upstream)	68	57–59

a Pol III promoters were selected from several organisms and cloned into a luciferase reporter to analyze promoter activity during infection. Parenthetical additions specify promoter features (3A, triplicated A box; for EBERs, + upstream includes upstream elements, no upstream excludes upstream elements).

**FIG 2:**
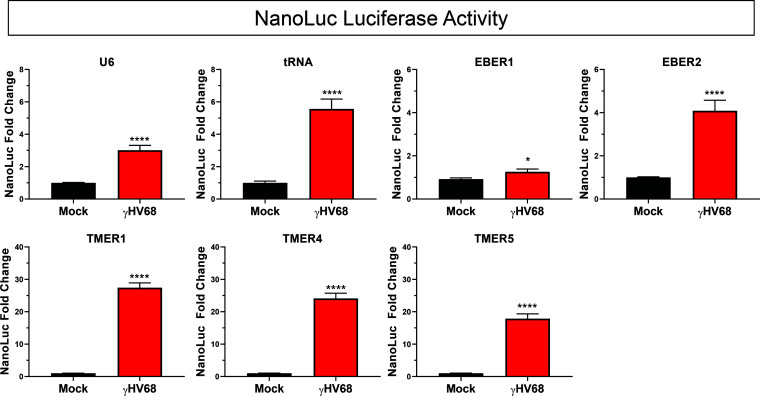
γHV68 infection induces activity of multiple pol III promoter types. Promoter activity was measured for the U6 (*n* = 8), tRNA (*n* = 3), EBER1 (*n* = 3), EBER2 (*n* = 4), TMER1 (*n* = 18), TMER4 (*n* = 4), and TMER5 (*n* = 6) promoters during infection. Luciferase assays were performed as previously described, with cell lysates collected 24 h postinfection. Each independent experiment (n) contained biological triplicates or duplicates. All promoter activity changes were analyzed as the fold change in NanoLuc activity normalized to uninfected samples (infected NanoLuc LU/mock NanoLuc LU). Error bars, SEM. Significant differences were analyzed by *t* test and are indicated as asterisks. *, *P* ≤ 0.05; ****, *P* ≤ 0.0001.

We further analyzed the activity of several other promoter types to assess how they are impacted during γHV68 infection. Experiments indicated that γHV68 infection induced activity from multiple pol III promoters, including the human U6 and tRNA-Tyr promoters, the EBER1 and EBER2 promoters, and TMER1, -4, and -5 promoters (Fig. 2). Although the vault RNA1-1 and adenovirus VA1 promoters were cloned into the reporter, there was no detectable activity from these constructs (unpublished data). While viral infection induced luciferase activity from all of the examined promoters, the TMER promoters consistently showed the greatest induction during infection.

**FIG 3:**
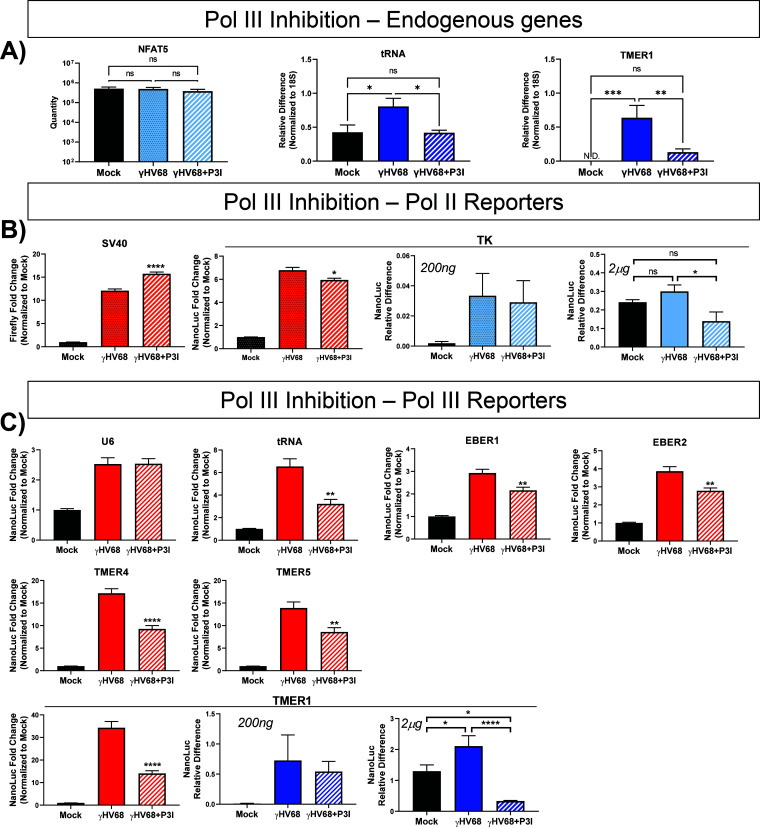
Inhibition of RNA polymerase III decreases luciferase activity and RNA levels from pol III promoter during γHV68 infection. Expression of endogenous control genes and reporter constructs were measured during treatment with a pol III inhibitor. Prior to transfection, samples were treated with an RNA polymerase III inhibitor (CAS 577784-91-9), marked as P3I, or left untreated. Cells were cotransfected with the SV40 promoter-driven Firefly luciferase control and NanoLuc reporter constructs with the indicated promoters. Inhibited cells were treated again immediately following infection. Luciferase activity is shown in orange for pol II promoters and red for pol III promoters. RNA expression is shown in light blue for pol II promoters and dark blue for pol III promoters. (A) Efficacy of pol III inhibition was shown by TaqMan RT-qPCR of a pol II-transcribed gene (NFAT5) and SYBR green qPCR of pol III-transcribed genes (human pretRNA-Tyr-GTA-1-1 and γHV68 TMER1). Data are from 3 independent experiments with biological duplicates or triplicates per experiment. Luciferase assays were performed as previously described, with cell lysates collected 24 h postinfection. Luciferase activities of the pol II promoters (orange, SV40-Firefly and TK-NanoLuc) (B) and pol III promoter-NanoLuc (red) (C) are shown. All luciferase activity changes are analyzed as the fold change in NanoLuc activity normalized to uninfected samples (infected NanoLuc LU/mock NanoLuc LU). NanoLuc RNA was also measured from a pol II promoter (light blue, TK) (B) and a pol III promoter (dark blue, TMER1) (C). RT-qPCR was performed for NanoLuc RNA as previously described. Cells were treated as described above and transfected with 200 ng or 2 μg of DNA. RNA was collected 24 hpi and analyzed for expression of NanoLuc RNA. Firefly activity from SV40 was measured from two independent experiments with eight samples per experiment. RT-qPCR data for 200 ng are from two independent experiments with biological triplicates, and data for 2 μg are from three independent experiments with biological triplicates or duplicates. Error bars, SEM. Significant differences were analyzed by *t* test and indicated as asterisks. *, *P* ≤ 0.05; **, *P* ≤ 0.01; ***, *P* ≤ 0.001; ****, *P* ≤ 0.0001.

Because the normal role of pol III is in transcription of noncoding RNAs, we wanted to directly measure the impact of a pol III-specific inhibitor (CAS 577784-91-9) on luciferase activity from the TMER1 promoter compared to pol II promoters (TK-NanoLuc and SV40-Firefly). We treated cells with 40 μM CAS 577784-91-9, a drug concentration previously reported in investigation of γHV68 induction of SINE RNAs (33), prior to transfection and infection. We found that inhibition of pol III with this drug concentration significantly reduced the induction of luciferase activity from the TMER1 promoter without toxicity (unpublished data) and reduced expression of endogenous pol III-transcribed genes during infection (Fig. 3A, human pretRNA-Tyr-GTA-1-1 and γHV68 TMER1). Pol III inhibition did not affect an endogenous pol II-transcribed gene (Fig. 3A, NFAT5) and had no consistent effect on the luciferase activity from pol II promoters SV40 and TK (Fig. 3B). In contrast, pol III inhibition led to significant decreases in luciferase activity from six of the seven tested pol III promoters (Fig. 3C), with the exception being the U6 promoter (the only type 1 promoter), which exhibited no decrease in activity. The apparent resistance of the U6 promoter to inhibition could be due to either the relatively high activity of the U6 promoter or alternate mechanisms of transcription at the U6 promoter (e.g., pol II recruitment to the promoter [41]) invoked at these inhibitor concentrations. Although these data show pol III activity was not completely inhibited, we expect that full inhibition of pol III would result in cell death; therefore, partial pol III inhibition is ideal for measuring the impact of pol III in this system. These studies indicate that inhibiting pol III activity consistently impaired induction of luciferase from multiple host and viral pol III promoters corresponding to the type 2 pol III promoter subtype.

We also measured the luciferase RNA expressed from the TK (pol II) and TMER1 (pol III) promoters during infection with pol III inhibition. When mimicking the transfection conditions used for the dual-luciferase assays with 200 ng transfected DNA, we found no significant difference in the expression of NanoLuc from either the TK or TMER1 promoters under any of the examined conditions (Fig. 3B and C, blue). However, given that there is induction of NanoLuc RNA from the TMER1 promoter during infection (see Fig. 5), we tested whether increasing the amount of transfected reporter DNA would allow for greater distinction in NanoLuc RNA levels under infection and pol III inhibition conditions. Thus, we repeated these experiments with 2 μg of total transfected DNA. These experiments showed no difference in NanoLuc expressed from the TK promoter during infection or pol III inhibition compared to mock infection. However, there was a significant decrease in NanoLuc expressed from the TMER1 promoter during infection with pol III inhibition compared to both mock infection (4-fold) and infection (6-fold). These data suggest that the NanoLuc expression from pol III promoters is dependent on pol III activity.

**FIG 4:**
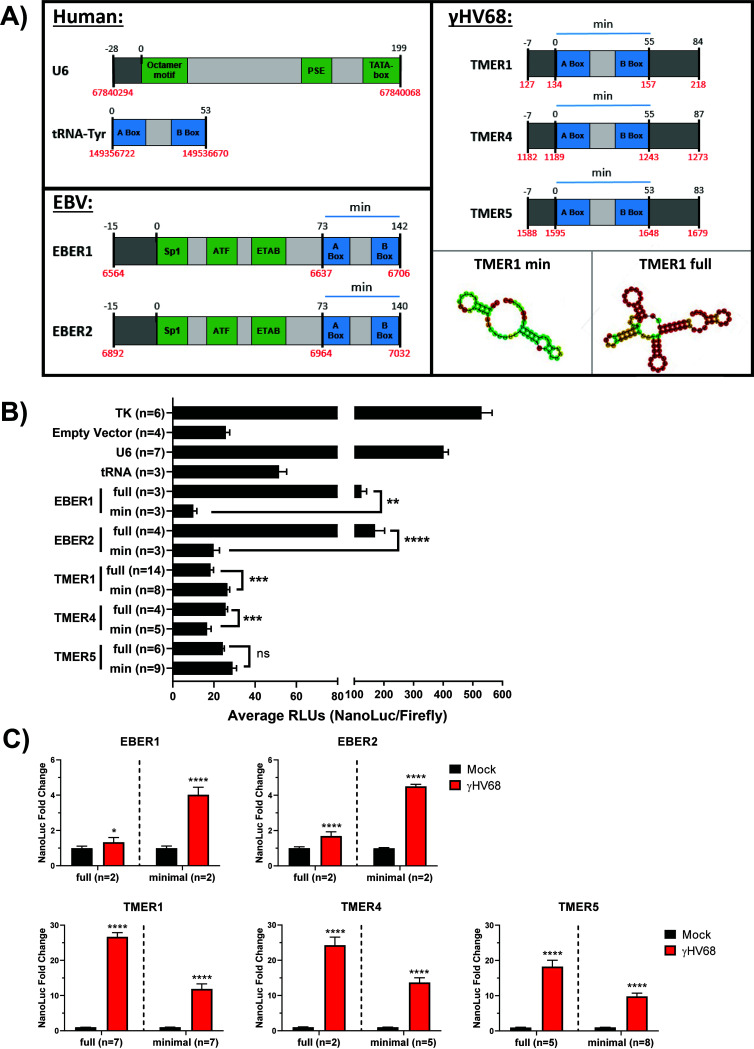
Sequence external to the minimal promoter elements alters promoter activity. (A) Several pol III promoters were cloned into the pNLP3 NanoLuc-expressing vector. Promoter schematics are not to scale. The canonical minimal promoter elements for each promoter are shown in blue and notated with a blue line labeled “min.” and the intermediate sequence in light gray. Upstream elements are green, and external/extended sequence is shown in dark gray. Nucleotide positions are shown above each schematic in relation to position 0, which indicates the start of the minimal promoter sequence. Genomic coordinates are shown below each schematic in red and refer to the following reference sequences: human U6 (RNU6-1), NC_000015.10, human tRNA-Tyr (TRY-GTA11-1), NC_000007.14, EBERs in human herpesvirus 4/EBV whole genome, NC_007605.1, TMERs in MHV68 whole genome, NC_001826.2. The coordinates for human U6 snRNA and tRNA are reversed to reflect their orientation as coded on the complement strand. The predicted structure for the minimal and full TMER1 promoter using the RNAfold Webserver are shown. (B) Cloned reporter constructs were transfected into HEK293 cells at a 10:1 molar ratio with a Firefly luciferase control expressed by the SV40 promoter. TK-expressed NanoLuc was used as a positive control. Cells were not infected. Following 48 h of transfection, dual-luciferase assays were performed as previously described. Luciferase activity is expressed as the ratio of NanoLuc activity to control Firefly activity (relative luminescence units, RLUs). (C) Luciferase assays were performed as previously described, with cell lysates collected 24 h postinfection. All promoter activity changes are analyzed as the fold change in NanoLuc activity normalized to uninfected samples. Each experiment (*n*) included biological triplicates. Error bars, SEM. Significant differences were analyzed by *t* test and indicated as asterisks. *, *P* ≤ 0.05; **, *P* ≤ 0.01; ***, *P* ≤ 0.001; ****, *P* ≤ 0.0001.

Our analyses, to this point, indicated that the TMER promoters expressed the highest induction in luciferase activity during infection; therefore, we compared the sequences of TMER promoters to identify which features of these promoters could contribute to this strong induction. The initially analyzed TMER promoters contained the TMER promoter as well as extended sequence around the minimal promoter elements (Fig. 4A). Considering that the extra sequence included in these “full” promoters may contribute to infection-induced activity, we created a panel of “minimal” TMER and EBER promoters that contain only the minimal RNA pol III promoter elements, i.e., the sequence beginning from the A box to the end of the B box (Fig. 4A). We initially compared the activity of these promoters under basal (no infection) conditions to calculate the average RLUs (NanoLuc LU/Firefly LU) in the absence of virus-induced changes. This analysis showed that U6 was the most active of all pol III promoters under uninfected conditions, followed by the full EBER promoters (Fig. 4B). In contrast, minimal EBER promoters showed a significant reduction in baseline luciferase activity. All TMER promoters (full or minimal) appeared similar to the empty vector, that is, to have virtually no activity under uninfected conditions. We then compared the induction of NanoLuc luciferase activity between the EBER and TMER minimal and full promoters during infection to determine the role of extended sequence on promoter activity. As previously described, these constructs were transfected into HEK 293 cells and then infected with γHV68. The fold change in NanoLuc activity relative to mock-treated samples was compared after 24 h of infection (Fig. 4C). When we compared the relative inducibility of EBER full versus minimal promoters, minimal promoters showed greater virus inducibility. This enhanced inducibility of the minimal EBER promoters likely reflects the reduced basal luminescence from these promoters (Fig. 4B). Conversely, TMER minimal promoters displayed a weaker induction during infection than their full counterparts, suggesting the sequence surrounding the TMER minimal promoters drives stronger expression during infection. These results indicate that the sequence surrounding minimal pol III promoter elements impacts both the basal activity and inducibility of these promoters during infection.

**FIG 5:**
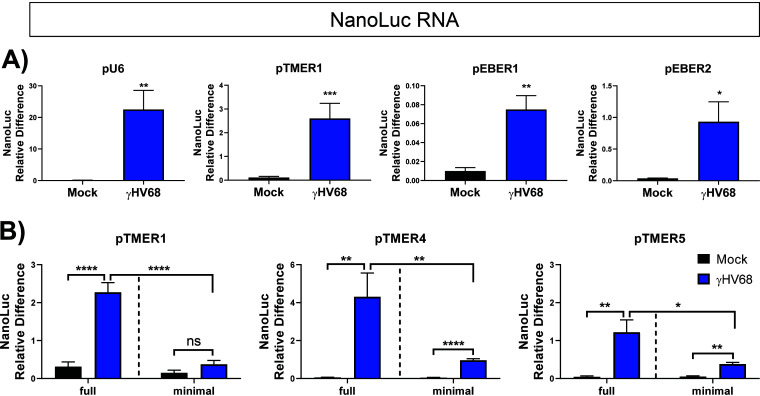
Effect of γHV68 infection on NanoLuc transcript levels. Promoter activity was measured for the U6, TMER1, and EBER full promoters (A) or the TMER1, TMER4, and TMER5 minimal versus full promoters (B). Cells were treated as previously described, and RNA was purified from cells at 16 h postinfection (U6, *n* = 2; TMER1, *n* = 5) or 24 hpi (EBER1, *n* = 2; EBER2, *n* = 2; TMER4, *n* = 2; TMER5, *n* = 2). RNA was converted to cDNA, and then LightCycler real-time PCR using Sybr green was performed with primers targeting the NanoLuc gene and a host control gene (18S). The relative difference of NanoLuc was calculated using the Pfaffl method (53), where the ratio is determined by (*E*_target_)^ΔCP target (control-sample)^/(*E*_ref_)^ΔCP ref (control-sample)^. Each experiment (*n*) included biological triplicates or duplicates. Error bars, SEM. Significant differences were analyzed by *t* test and are indicated as asterisks. *, *P* ≤ 0.05; **, *P* ≤ 0.01; ***, *P* ≤ 0.001; ****, *P* ≤ 0.0001.

Luciferase readouts of pol III promoter activity allowed us to uniformly analyze pol III promoter activity. This assay does not directly measure the level of RNAs, however, instead relying on an enzymatic readout of luciferase protein activity. To ensure that γHV68 infection was inducing pol III activity transcriptionally, we used the same NanoLuc constructs to measure promoter activity at the RNA level by performing RT-qPCR for the NanoLuc transcript. By following the same protocol as that used for the luciferase assays, HEK 293 cells were transfected with pGL3 and the pNLP3 vector expressed by pol III promoters of interest (as outlined in Fig. 1). Cells were then infected with γHV68, and RNA was purified from cells 16 or 24 h postinfection (hpi). Primers targeting the NanoLuc gene were used for qPCR following reverse transcription of the RNA. Infection increased the NanoLuc RNA expression from the U6 and TMER1 promoters, with more modest induction from the EBER promoters (Fig. 5A). These results indicate that γHV68 infection stimulates pol III promoter activity from multiple host and viral promoters, measured at both the transcriptional and translational levels. To extend these findings, we further measured NanoLuc RNA expression from minimal or full TMER promoters. These studies demonstrated that γHV68 infection increased NanoLuc RNA from the minimal promoter relative to mock-infected samples, with further RNA induction from the full TMER promoter (Fig. 5B). These results strongly suggest that the NanoLuc reporter assay serves as a faithful readout for pol III-dependent transcription, quantified at both the RNA and protein level. These findings also emphasize that sequences outside the minimal TMER promoters contribute to increased expression during infection.

**FIG 6:**
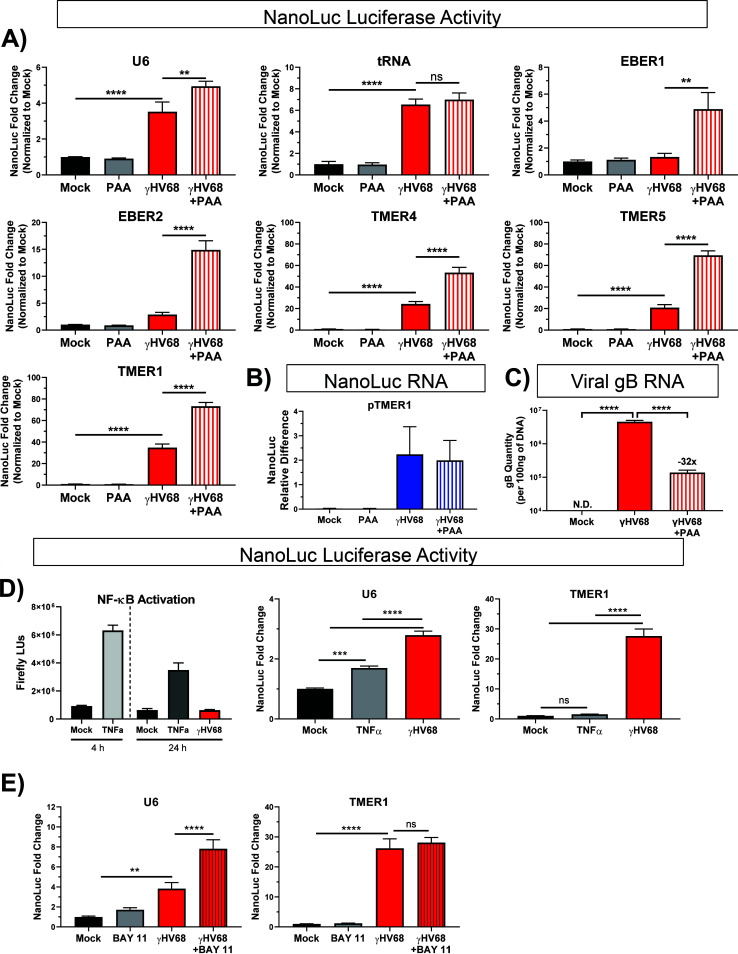
Effect of viral and NF-κB inhibitors and an NF-κB activator on luciferase activity during γHV68 infection. (A) Promoter activity was measured via luciferase activity for the following promoters during infection, including with concurrent PAA treatment: TMER1 (*n* = 5), TMER4 (*n* = 2), TMER5 (*n* = 2), EBER1 (*n* = 2), EBER2 (*n* = 3), U6 (*n* = 2), and tRNA (*n* = 1). Each experiment contained biological duplicates or triplicates. Luciferase assays were performed as previously described, with cell lysates collected 24 h postinfection. NanoLuc fold change for infected samples is from previous figures for comparison to PAA-treated samples. All promoter activity changes are analyzed as the fold change in NanoLuc activity normalized to uninfected samples. (B) Promoter activity was measured via RT-qPCR of the NanoLuc transcript expressed from the TMER1 promoter during infection and concurrent phosphonoacetic acid (PAA) treatment. RT-qPCR was performed as previously described. Data are shown as NanoLuc relative difference to a host gene (18S). *N* = 2 with biological triplicates. (C) Using the same RNA from experiments shown in panel B, RT-qPCR was performed for a viral late gene (gB) to confirm the efficacy of PAA treatment, which resulted in an approximately 32-fold decrease in gB compared to WT infected samples. gB was not detected (N.D.) in mock samples. (D) NF-κB activation with 50 ng/μl TNF-α (NF-κB reporter, *n* = 1 for each time point; U6, *n* = 2; TMER1, *n* = 3). (E) NF-κB inhibition by 10 μM BAY 11–7082 (U6, *n* = 3; TMER1, *n* = 4). NF-κB activation is shown in Firefly luminescence units, while NanoLuc activity from the U6 or TMER1 promoters is expressed as fold change over mock infection. Cells included in the TNF-α experiments were transfected for 12 h, while all others were transfected for 24 h as previously described. Each experiment (*n*) contains biological triplicates. Error bars, SEM. Significant differences were analyzed by *t* test (two conditions) or one-way ANOVA (three conditions) and are indicated as asterisks. *, *P* ≤ 0.05; **, *P* ≤ 0.01; ***, *P* ≤ 0.001; ****, *P* ≤ 0.0001.

γHV lytic replication critically depends on viral DNA replication and late gene transcription, processes that are inhibited by phosphonoacetic acid (PAA) (42, 43). Therefore, we tested the impact of PAA on virus-induced pol III induction. To do this, HEK 293 cells were transfected with the pol III-driven NanoLuc constructs and infected as before, with one set of samples receiving PAA treatment (200 μg/ml) after 1 h of viral inoculation. PAA treatment was consistently associated with increased luciferase enzymatic activity, with PAA-treated γHV68-infected cultures characterized by a greater apparent induction of luciferase activity than γHV68-infected cultures alone. This PAA-driven enhancement of luciferase activity was observed for multiple pol III promoters, including U6, TMER1, -4, and -5 and EBER1 and -2 (Fig. 6A). To determine if this effect was also observed at the transcriptional level, cells were transfected and infected as before. RNA was isolated 16 h postinfection, and RT-qPCR was performed to detect the NanoLuc transcript. Notably, treatment with PAA during γHV68 infection had no impact on the induction of NanoLuc RNA compared to untreated infected cells, indicating that viral DNA replication and late gene synthesis was not required for pol III induction (Fig. 6B). Effectiveness of the PAA treatment was confirmed by measuring expression of a viral late gene, gB (Fig. 6C). The increase in luciferase activity following PAA treatment, with minimal impact on NanoLuc RNA, strongly suggests that PAA treatment enhanced the translational output from the promoters tested. These data suggest that viral late gene expression plays an additional role in translation that is not seen at the transcriptional level, a phenomenon independent of pol III promoter activity.

Given the reported relationship between the NF-κB pathway and the expression of pol III-dependent transcripts (30), we analyzed the effect of NF-κB activation or inhibition on the activity of the U6 and TMER1 promoters via luciferase activity. First, we measured induction of an NF-κB reporter plasmid following treatment with either tumor necrosis factor alpha (TNF-α), a known inducer of the NF-κB pathway, or following γHV68 infection. Whereas TNF-α induced NF-κB reporter activity at 4 and 24 h posttreatment, γHV68 infection had no measurable impact on expression from the NF-κB reporter (Fig. 6D). Next, we analyzed the impact of NF-κB manipulation on pol III promoter activity. Treating cells with TNF-α modestly increased U6 promoter activity, albeit to a lesser extent than γHV68 infection, while TMER1 promoter activity was not affected by TNF-α treatment (Fig. 6D). Inhibition of NF-κB with the BAY 11–7082 (BAY 11) compound increased U6-expressed luciferase activity under virus-infected conditions but had no significant impact on TMER1 promoter activity after infection (Fig. 6E). This indicates a role of NF-κB in inhibiting pol III promoter activity during infection; however, this effect is only observed in the case of a gene-external (i.e., type 3) promoter. Ultimately, these data do not support a significant role of the NF-κB pathway in the observed induction of pol III promoter activity after γHV68 infection under these culture conditions.

**FIG 7:**
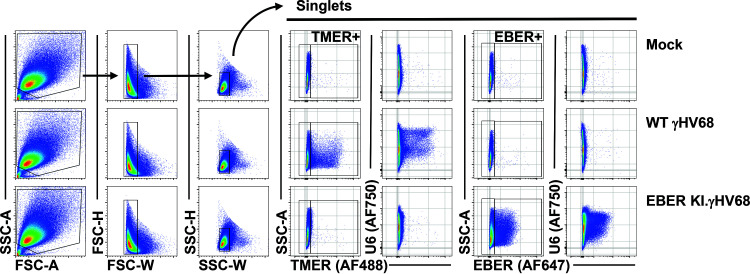
Viral ncRNA expression serves as an indicator of virus infection. Murine 3T12 fibroblasts were mock infected or infected with WT γHV68 or an EBER knock-in (EBER-KI) γHV68 at an MOI of 1, harvested at 16 hpi, and subjected to the PrimeFlow RNA assay and flow cytometric analysis. Singlets were identified using a sequential gating strategy as shown, with representative examples from mock (top)-, WT γHV68 (middle)-, or EBER-KI.γHV68-infected samples. Singlet populations were then analyzed for TMERs (type 4/AF488), EBERs (type1/AF647), and U6 snRNA or 4.5S RNA (type 6/AF750 for both, each in different probe mixes).

**FIG 8:**
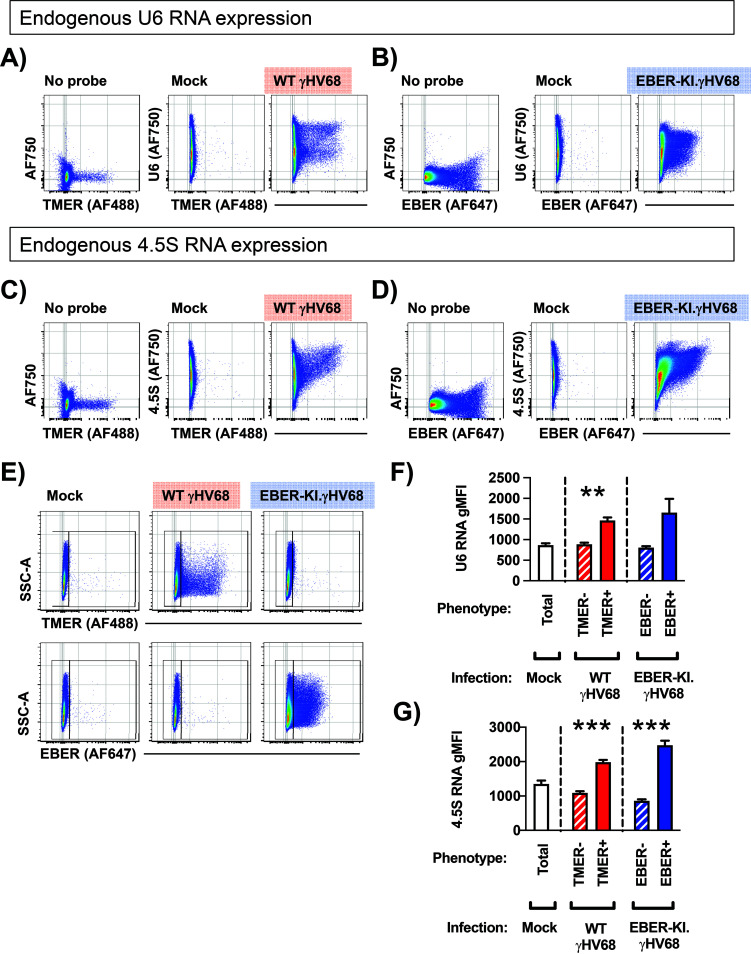
Endogenous expression of murine U6 snRNA and 4.5S rRNA during γHV68 infection. Murine fibroblast cells (3T12) were mock infected or infected with WT γHV68 or an EBER knock-in (EBER-KI) γHV68 at an MOI of 1, harvested at 16 hpi, and subjected to the PrimeFlow RNA assay and flow cytometric analysis. (A and B) Analysis of U6 snRNA expression comparing background fluorescence (No probe, left) versus U6 probe hybridization in mock (middle)-, WT γHV68-, or EBER-KI.γHV68-infected cells (right). (C and D) Analysis of 4.5S rRNA expression comparing background fluorescence (No probe, left) versus 4.5S rRNA probe hybridization in mock (middle)-, WT γHV68-, or EBER-KI.γHV68-infected cells (right). (E) Identification of virally infected cells that express either the TMERs or the EBERs following mock infection or infection with WT γHV68 or EBER-KI.γHV68. Gates define cells based on positive or negative expression as indicated, with these populations used for subsequent quantitation in panels F and G. (F and G) The geometric mean fluorescence intensity (gMFI) was calculated for U6 snRNA (F) or 4.5S rRNA (G) in mock-infected or virus-infected samples, comparing cells that differed in expression of either the TMERs or the EBERs in WT or EBER-KI infections, respectively. Data depict flow cytometric analysis of singlets, defined by sequential discrimination using SSC and FSC parameters as shown in Fig. 7. Data are from 3 biological replicates, with statistical significance assessed using an unpaired *t* test. **, *P* < 0.01; ***, *P* < 0.001.

The NanoLuc-expressing constructs allowed us to measure promoter activity using a reporter system, including a shared readout that minimizes confounding factors of RNA sequence/structure/stability, and our analysis of pol III promoter activity suggested a general induction during infection. However, a previous report suggested that only a subset of host pol III-transcribed genes, the SINE RNAs, are increased during lytic γHV68 infection of NIH 3T3 cells and *in vivo* infections of C57BL/6 mice (33). While our reporter studies rely on cell systems that can be easily transfected with reporter DNA, we wanted to quantify the impact of γHV68 infection on the abundance of endogenous murine and viral pol III RNAs yet avoid challenges in PCR amplification, unique primer/probe designs, and bulk analysis. To accomplish this, we made use of the PrimeFlow assay system, a sensitive and robust fluorescent *in situ* hybridization assay, combined with multiparameter flow cytometry. This system quantifies steady-state RNA expression at the single-cell level using direct probe hybridization to endogenous RNAs, with sensitivity provided by amplification based on probe stacking rather than PCR and primers. For this analysis, murine fibroblast cells (3T12) were mock treated, infected with wild-type (WT) γHV68, or infected with an EBER-knock-in (EBER-KI.γHV68) recombinant γHV68 that lacks the TMERs and instead contains insertion of the EBV EBERs into the TMER locus. EBER-KI.γHV68 was competent for viral replication (unpublished data). 3T12 cells were infected at a multiplicity of infection (MOI) of 1 and harvested at 16 h postinfection, conditions that result in a mixed population of virally infected and uninfected cells. Cells were then queried with fluorescent probes to detect viral (the γHV68 TMERs or the EBV EBERs) and host ncRNAs (U6 snRNA or 4.5 rRNA, the murine equivalent of human 5S rRNA). This analysis allowed a comparison of host ncRNA expression as a function of viral ncRNA expression, comparing cells with or without virus ncRNA expression. Gating schemes for doublet exclusion and probe specificity are shown in Fig. 7. Notably, probes for the TMERs and EBERs were specific for their intended targets, with no detectable TMER or EBER signal in mock-infected samples, TMER^+^ cells present only in WT γHV68-infected samples, and EBER^+^ cells present only in EBER-KI.γHV68-infected samples (Fig. 8E). We next compared the relative expression of the U6 (Fig. 8A and B) and 4.5S ncRNAs (Fig. 8C and D) between cells with active viral RNA expression and cells that did not express these viral ncRNAs. Analysis of the geometric mean fluorescence intensity (gMFI) for the U6 snRNA and 4.5 rRNA probes revealed increased expression of U6 and 4.5 rRNA ncRNAs in virally infected cells (i.e., TMER^+^ or EBER^+^) compared to uninfected cells (TMER^−^ or EBER^−^) (Fig. 8F and G). These data demonstrate an increase in host pol III-transcribed ncRNAs in γHV68-infected cells and emphasize the benefit of single-cell analysis to quantify endogenous ncRNA expression during virus infection.

**FIG 9:**
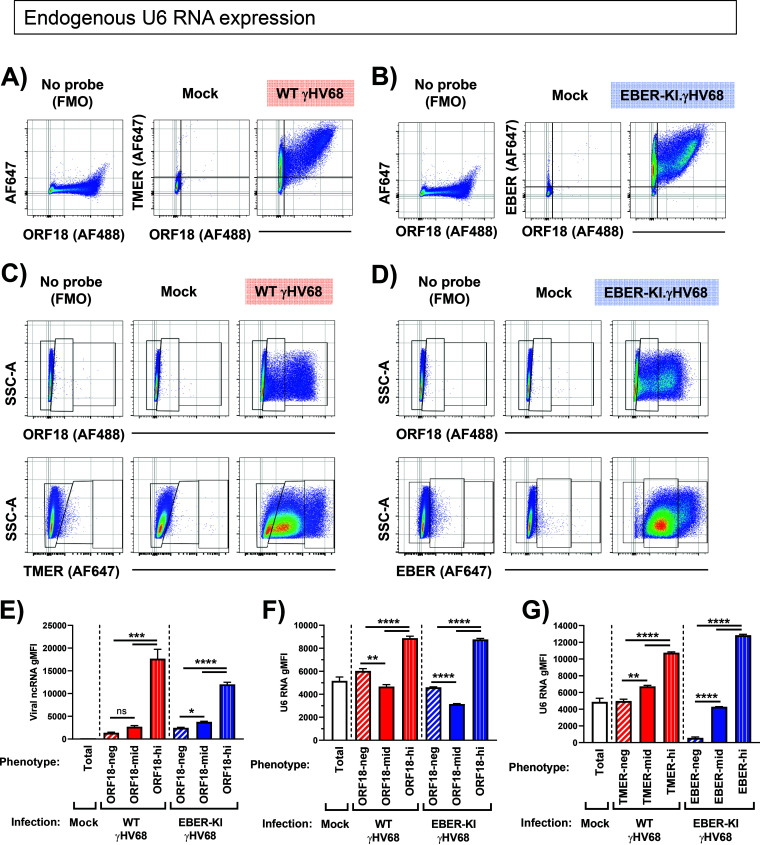
Endogenous expression of murine U6 snRNA during γHV68 infection. Murine fibroblast cells (3T12) were mock infected or infected with WT γHV68 or an EBER knock-in (EBER-KI) γHV68 at an MOI of 5, harvested at 16 hpi, and subjected to the PrimeFlow RNA assay and flow cytometric analysis. (A and B) Analysis of viral gene expression indicating viral ncRNA (TMER or EBER) expression and/or ORF18 expression comparing background fluorescence (No probe, left) versus TMER or EBER hybridization in mock (middle)- or WT γHV68- or EBER-KI.γHV68-infected (right) cells. (C and D) Analysis of viral gene expression indicating ORF18 expression (top) or viral ncRNA (TMER or EBER, bottom) expression comparing background fluorescence (No probe, left) to probe hybridization in mock (middle)-, WT γHV68 (C)-, or EBER-KI.γHV68r (D)-infected cells (right). These plots indicate the gating used for further analysis in panels E and F. (E) The geometric mean fluorescence intensity (gMFI) was calculated for viral ncRNA (TMERs or EBERs) in mock-infected or virus-infected samples, comparing cells that differed in expression of ORF18. (F and G) The gMFI was calculated for U6 snRNA in mock- or virus-infected samples, comparing cells that differed in expression of either ORF18 (F) or TMERs or EBERs (G) in WT or EBER-KI infections. Data depict flow cytometric analysis of singlets, defined by sequential discrimination using SSC and FSC parameters, as exemplified in Fig. 7. Data are representative of two experiments, with 3 biological replicates in each. Statistical significance assessed using a one-way ANOVA with multiple comparisons. *, *P* ≤ 0.05; **, *P* ≤ 0.01; ***, *P*≤ 0.001; ****, *P* ≤ 0.0001.

This analysis established a correlation between expression of viral and host ncRNAs at the single-cell level but raised the potential that detection of endogenous viral ncRNAs could bias our analysis to cells with higher pol III activity independent of viral effects. To address this concern, we repeated the analysis with another, pol III-independent readout of infection, detection of the pol II-dependent γHV68 ORF18 RNA. Cells were treated as before, with the modification that they were infected at an MOI of 5 rather than 1, so that differences between infected and noninfected cells were exaggerated. To simplify the RNA probe panel and to fortify previous data, we focused on the U6 snRNA as a host measure of pol III activity. Infected cells were defined as ORF18 RNA positive as well as TMER (Fig. 9A) or EBER (Fig. 9B) positive for WT and EBER-KI γHV68 infection, respectively. This analysis identified several populations that could be distinguished by different expression levels (negative, mild, or high expression) for either ORF18 or the viral ncRNAs (Fig. 9C and D). Within each ORF18-defined population, we calculated the gMFI for either the TMERs/EBERs (Fig. 9E) or U6 snRNA (Fig. 9F). ORF18 RNA^high^ cells had the highest expression of the TMERs in WT-infected samples and the EBERs in EBER-KI-infected samples compared to ORF18 RNA^mid^ or ORF18-negative cells (Fig. 9E). Notably, virally infected ORF18 RNA^high^ cells from either WT or EBER-KI γHV68 infection were also characterized by increased U6 expression relative to uninfected, ORF18-negative cells in the same cultures (Fig. 9F). Consistent with our previous observations, U6 RNA gMFI was highest in cells with highest expression of either the TMERs or EBERs (Fig. 9G). These data demonstrate that virally infected cultures are characterized by variation in ncRNA expression between individual cells and that cells with abundant virus transcription are further characterized by increased host and viral ncRNA expression.

## DISCUSSION

While γHV infection is known to alter expression of host genes and viral ncRNAs are integral for pathogenesis, the transcriptional regulation of these ncRNAs has remained unclear. Here, we propose that different pol III promoter types allow for distinct means for transcriptional regulation during infection. This study focused on the impact of γHV68 lytic replication on pol III promoter activity. To measure pol III activity, we used three highly sensitive methods, two of which were based on a reporter gene and one method based on measuring authentic RNAs. Although these methods each have different advantages and disadvantages, they all indicate that lytic infection drives a general upregulation of promoter activity across multiple host and viral pol III-dependent transcripts, as shown by upregulation of both luciferase activity and luciferase RNA expression as well as increased expression of endogenous transcripts, as measured by flow cytometric analysis of pol III RNAs. Our studies further reveal distinct effects of specific promoters and promoter features. These findings emphasize the utility of the modified NanoLuc luciferase system to analyze pol III promoter activity and provide clear evidence for pol III promoters with large differences in basal and inducible promoter activity. They further emphasize the capacity of γHV lytic infection to modify pol III-dependent transcriptional machinery in infected cells, a process that likely facilitates productive virus replication (33). At this time, it remains unknown whether pol III machinery or transcription is altered during γHV68 latent infection or reactivation from latency.

Our use of a luciferase reporter to measure the activity of pol III promoters allowed us to directly compare the functional activity of multiple pol III promoters in a high-throughput assay while minimizing potential differences that may arise due to ncRNA sequence, structure, or stability. While these assays used NanoLuc RNA and protein as a standard platform for analysis, an important caveat of this analysis is that many pol III promoters are gene-internal. Based on this, the resulting transcript is comprised of a hybrid of at least the minimal pol III promoter elements (i.e., the A and B box) fused directly upstream of the NanoLuc RNA, creating a hybrid RNA that is not 100% identical between constructs. Although pol III promoters conventionally drive expression of noncoding RNAs, there is clear precedent that pol III can transcribe translation-competent RNA (44, 45), and luciferase reporters have been used for high-throughput and unbiased analysis of pol III promoter activity (38, 46). Inspired by these studies, we cloned several host and viral ncRNA promoters into a NanoLuc luciferase reporter to measure their activity during lytic γHV68 infection. We chose the NanoLuc luciferase as our reporter due to the small size and high activity, which is consistent with pol III transcriptional capacity and measurement of genes that may be expressed at a low level. To further enhance the robustness of this reporter, we identified and removed a pol III termination sequence within the NanoLuc gene that approximately doubled luciferase reporter activity. Although pol III transcription theoretically should be terminated in the original NanoLuc reporter, pol III readthrough of termination signals has been reported (47). In total, use of the modified NanoLuc reporter construct afforded a sensitive and robust readout for assessing pol III promoter activity.

Through use of this pol III reporter assay, we found that γHV68 infection increased promoter activity across a range of host and viral pol III promoters, as measured by both luciferase activity and reporter RNA abundance. Pol III transcription from these promoters was confirmed through treatment with a pol III-specific inhibitor (CAS 577784-91-9). These results demonstrate that virus induction of pol III promoters is dependent on RNA polymerase III, either through direct or indirect means. Interestingly, the consequence/magnitude of induction elicited by infection varied between promoters. For example, the U6 promoter conveyed high basal activity, with infection resulting in a modest induction of U6 promoter activity. Conversely, the TMER promoters exhibited extremely low basal activity under mock-infected conditions, with dramatic induction after γHV68 infection. The inducibility of the TMERs was further enhanced by accessory sequences outside the minimal A and B box elements. One explanation for this enhanced induction is that these extended sequences may contain additional transcriptional elements that are integral to the promoter itself. While formally possible, it is notable that accessory sequences across the TMERs are not conserved (6, 22, 23). An alternate explanation for the enhanced activity of the full TMER promoters is that inclusion of the extended sequence includes the full tRNA-like structure of the TMER genes (Fig. 4A). It is interesting to speculate that this tRNA-like structure could either lend greater stability to transcripts or protect the transcripts from degradation by host exonucleases or the viral endonuclease, muSOX (48). Although pol III transcription is frequently associated with the transcription of housekeeping ncRNAs, there is clear precedent that pol III can also participate in inducible gene expression (e.g., γHV68-induced expression of SINEs) (33, 34). Whether the TMERs have conserved regulatory mechanisms with host-inducible ncRNAs is currently unknown; however, the TMERs share promoter similarity to the SINEs (type 2, gene internal).

Our studies demonstrated that infection increased NanoLuc expression at both the RNA and protein activity level, indicating that virus infection increased pol III promoter activity and not some secondary measurement. While we saw broad induction of luciferase activity across pol II and pol III promoters, induction of activity from pol III promoters was inhibited by a pol III inhibitor, consistent with a model that these promoters are recruiting pol III to transcribe translation-competent RNA. Notably, induction of the reporter RNA, as measured by RT-qPCR, was specific to a pol III promoter (TMER1), and no significant induction was seen from a pol II promoter (TK, Fig. 3B). This suggests caveats in the induction we measure from luciferase activity versus luciferase RNA. A difference in reporter protein activity versus transcript abundance was also seen when we inhibit viral late gene expression. Unexpectedly, inhibition of viral late gene expression with PAA resulted in increased luciferase activity from nearly all of the promoters examined. This phenomenon was only seen at the level of NanoLuc protein activity, not at the level of NanoLuc RNA. The ability of PAA to enhance NanoLuc protein activity, with no commensurate change in RNA expression, suggests that viral late genes have a role in tempering translation. Together with the general induction of luciferase activity seen during infection, these data suggest that viral induction of gene expression is occurring at both the transcriptional and translational levels. Infection may induce expression of pol III-derived reporter transcripts (RNA) and also increase general translation (luciferase activity), leading to a compound effect when we measure reporter protein activity. Manipulation of host translational machinery by the herpesviruses is a common strategy that is required for optimal virus replication and the production of virus progeny (49).

While experiments with the NanoLuc reporter constructs indicated a general induction in the activity of the observed pol III promoters, the levels of induction varied by promoter type. Interestingly, promoters with upstream elements (U6, EBER1, and EBER2) displayed the highest level of basal activity under mock-infected conditions and the lowest level of virus-mediated induction of luciferase activity, while gene-internal type 2 promoters had the lowest basal activity and highest induction (Fig. 2). This is likely not due to promoters with gene external elements reaching the limit of detection of the luciferase assay, as demonstrated by the increased induction seen with PAA treatment (Fig. 6). Notably, the luciferase activity expressed from the U6 promoter was not significantly impacted by pol III inhibition, while all other pol III promoters showed decreased activity (Fig. 3C). Among the promoters with external elements, the U6 promoter has the highest induction of NanoLuc expression when measured at the RNA level, and the EBERs had a relatively low induction, with the gene-internal TMERs moderately induced (Fig. 5). The inhibitors used in this project also appear to have promoter-specific affects, with NF-κB modulation altering the output from the type 1 U6 promoters but not the type 2 TMER promoter (Fig. 6). Although all transcripts are transcribed by pol III, these data indicate various levels of induction of the different promoters, with various abilities for the resulting transcripts to be translated into functional proteins. As evidenced by the U6 promoter, the external elements of type 1 pol III promoters seem to drive unique responses compared to other pol III promoters, and these unique characteristics must be kept in mind when analyzing different promoter types.

Due to the possibility that viral infection has unique effects on transfected plasmids, we complemented our reporter studies with a highly sensitive analysis of endogenous and viral gene expression using the PrimeFlow assay to quantify endogenous RNAs by flow cytometry. Notably, this assay allowed us to identify gene expression differences within individual cells that may be lost in bulk analysis. Furthermore, the use of a branching fluorescent probe targeting the RNA of interest allows magnification of low signals without requiring replication of the gene of interest with potentially inadequate/inefficient primers. These data were consistent with our previous findings, indicating that viral infection leads to the induction of both viral (TMERs and EBERs) and host (U6 snRNA and 4.5 rRNA) pol III-derived transcripts. An alternate explanation for these findings is that cells with inherently higher pol III activity are more susceptible to viral infection, resulting in the observation of high viral pol II gene expression (ORF18) coupled with high expression of pol III-derived ncRNAs. As this assay measures steady-state RNA levels that are influenced by RNA production and decay, changes in measured RNA levels may be influenced by factors such as transcriptional induction, RNA stability, and/or degradation.

Previous reports show that γHV infection can have diverse effects on pol III transcription, ranging from a general induction of pol III machinery (e.g., in the context of EBV and EBNA1 [32, 50]) to the selective induction of pol III-transcribed RNAs (e.g., induction of specific host vault RNAs in EBV latently infected cells [30, 31] and the host SINE RNAs in γHV68-infected cells [33]). One challenge in interpreting these different findings is that these studies have been done in different states of infection (latent versus lytic), in different cell types, and in different states of cellular transformation. In many cases, the mechanistic insights gained from these studies could only be gained through the use of *in vitro* studies. In keeping with this, we anticipate that the γHV68 system will afford unique insights in how the γHVs regulate pol III-dependent ncRNA expression, allowing the analysis of primary infection coupled with technologies to measure promoter activity and endogenous ncRNA expression. For example, our single-cell analysis of pol III-derived transcripts, U6 snRNA and 4.5 rRNA, supported that γHV68 infection not only increases pol III-dependent promoter activity (as observed in experiments with reporter constructs) but also increases the endogenous expression of these transcripts. Future strategies to improve high-throughput direct comparison of promoters could include the use of reporter-based constructs such as those described in this study along with other reporter-based readouts, such as recently reported fluorescent RNA aptamers that do not rely on translation (51). How host and viral ncRNAs are regulated as a function of cell type and virus stage of infection remains an important unanswered question.

In total, our studies revealed a γHV68-dependent induction in the activity of host and viral pol III promoters. This induction was seen in the expression of a reporter gene as well as in the endogenous expression of pol III-dependent transcripts. Although previous reports focused on the virus-mediated upregulation of specific host ncRNAs, these experiments suggest a broader effect of lytic γHV68 infection on pol III activity. This suggests that γHV68 modulation of the host transcriptional landscape goes beyond mRNA regulation and that pol III-dependent transcripts are likely to play a wider role in γHV68 pathogenesis than previously appreciated.

## MATERIALS AND METHODS

### Viruses and tissue culture.

All viruses were derived from a bacterial artificial chromosome (BAC)-derived WT γHV68 (52). For some experiments, the TMER total knockout (TMER-TKO) virus was used; this virus was generated as previously described (25). Viruses were propagated and titers determined as previously described (25). EBER-KI virus contains EBERs 1 and 2 in the TMER-TKO virus backbone. Its generation and characterization will be described in a future work.

Human endothelial kidney (HEK 293) and murine fibroblast (3T12) cells were cultured in Dulbecco’s modified Eagle medium (DMEM; Life Technologies) supplemented with 5% fetal bovine serum (FBS; Atlanta Biologicals), 2 mM l-glutamine, 10 U/ml penicillin, and 10 μg/ml streptomycin sulfate (complete DMEM). Cells were cultured at 37°C with 5% CO_2_.

### Mutagenesis of pNL1.1 to create the pNLP3 NanoLuc luciferase reporter.

The promoterless NanoLuc luciferase reporter vector pNL1.1[*Nluc*] was obtained from Promega, and primers were designed to introduce silent mutations to remove the pol III termination signal in the NanoLuc coding sequence; these primers are listed in Table 2. Mutagenesis PCR was performed with the following cycles: (i) 95°C for 30 s, (ii) 12 cycles of 95°C for 30 s, 55°C for 1 min, and 68°C for 3 min. The resulting DNA was digested with DpnI (New England Biolabs Inc.) and transformed into XL1-Blue supercompetent cells (Agilent). Bacterial colonies were sequenced to confirm the correct mutations. The resulting plasmid was named pNLP3 to indicate that it is a NanoLuc plasmid optimized for pol III.

**TABLE 2:** Sequences of primers and oligonucleotides used in this study^*a*^

Name	Sequence	Cloning template	Purpose
NanoLuc Mut For.	5′-C CAA ATG GGC CAG ATC GAA AAA ATC TTC AAG GTG GTG TAC CC-3′	pNL1.1 (Promega)	Remove termination signal (TTTT) from pNL1.1 to make pNLP3
NanoLuc Mut Rev.	5′-GG GTC CAC CAC CTT GAA GAT TTT TTC GAT CTG GCC CAT TTG G-3′	pNL1.1 (Promega)	
U6-F-XhoI	5′-GTTATTCTCGAGGAGGGCCTATTTCCCATG-3′	SHC001 (Functional Genomics Facility)	Clone full U6 promoter into pNLP3 (product length, 227 bp)
U6-R-HindIII	5′-GCCGCCGAAGCTTATATATAAAGCCAAGAAATC-3′	SHC001 (Functional Genomics Facility)	
TMER-1-F-XhoI	5′-GTTGTTCTCGAGGCCAGAGTAGCTCAATTC-3′	γHV68 left-end plasmid	Clone TMER1 promoter into pNLP3 (product, 91 bp)
TMER-1-R-HindIII	5′-GTCGTTAAGCTTAGTTGGACCCACTTCCTC-3′	γHV68 left-end plasmid	
TMER4full_F_XhoI	5′-GTTATTCTCGAGGTCGGGGTAGCTCAATTG-3′	γHV68 left-end plasmid	Clone TMER4 promoter into pNLP3 (product, 93 bp)
TMER4full_R_HindIII	5′-GTTGTGAAGCTTGACGACCCGATCTCAAC-3′	γHV68 left-end plasmid	
TMER5full_F_XhoI	5′-GTACTACTCGAGGCCAGGGTAGCTCAATTG-3′	γHV68 left-end plasmid	Clone TMER5 promoter into pNLP3 (product, 94 bp)
TMER5full_R_HindIII	5′-GTTGTGAAGCTTTACCGCACCTCCAC-3′	γHV68 left-end plasmid	
EBER1-XhoI-F	5′-GTTGTTCTCGAGCAACTATAGCAAACCCCG-3′	pSP73-EBER plasmid	Clone full EBER1 promoter, including upstream elements, into pNLP3 (product length, 157 bp)
EBER1-HindIII-R	5′-GTTGTTAAGCTTGGGACTTGTACCCGGG-3′	pSP73-EBER plasmid	
EBER2-XhoI-F	5′-GTCGTTCTCGAGAGATGCACGCTTAACC-3′	pSP73-EBER plasmid	Clone full EBER2 promoter, including upstream elements, into pNLP3 (product length, 156 bp)
EBER2-HindIII-R	5′-GGTGTTAAGCTTGGGACTTGACCTCGG-3′	pSP73-EBER plasmid	
EBER1-GI-XhoI-F	5′-GTTCTTCTCGAGCGCTGCCCTAGAG-3′	pSP73-EBER plasmid	Clone EBER1-minimal promoter into pNLP3. Used with EBER1-HindIII-R, product, 70 bp
EBER2-GI-XhoI-F	5′-GTTGTTCTCGAGCGTTGCCCTAGTGGTTTC-3′	pSP73-EBER plasmid	Clone EBER2-minimal promoter into pNLP3. Used with EBER2-HindIII-R, product, 68 bp
NanoLuc_Forward	5′-CAC CAT GGT CTT CAC ACT CG-3′		RT-PCR on pNL1.1 and pNLP3 for the NanoLuc gene
NanoLuc-full_Rev	5′-CTA GAG TCG CGG CCT TAC G-3′		
NanoLuc-prestop_Rev	5′-CGA TCT GGC CCA TTT GGT C-3′		
Syber-NanoLuc-F	5′-CACTGGTAATCGACGGGGTT-3′		qPCR for the NanoLuc gene
Syber-NanoLuc-R	5′-TTTTGTTGCCGTTCCACAGG-3′		
pNL1.1_MCR_seq	5′-GTG TGA ATC GAT AGT ACT AAC ATA CGC-3′		Ideal sequencing of any insert in the MCR of pNL1.1 or pNLP3
5SrRNA-XhoI-F	5′-TCGAGAGCTAAGCAGGGTCGGGCCTGGTTAGTACTTGGATGGGAGAC-3′		Oligonucleotides annealed then enzyme-treated to create the 5S rRNA promoter, then inserted into pNLP3
5SrRNA-HindIII-R	5′-AGCTTGTCTCCCATCCAAGTACTAACCAGGCCCGACCCTGCTTAGCT-3′		
Ad2-VAI_Fwd	5′-GTT GTT CTC GAG GTG GTC TGG UGG ATA AAT TCG CAA GGG TAT CAT GGC GGA CGC CCG GGG TTC GAA CCC CAA GCT TGT CGT C-3′		Oligonucleotides annealed then enzyme-treated to create the adenovirus VA1 promoter, then inserted into pNLP3
Ad2-VAI_Rev	5′-GAC GAC AAG CTT GGG GTT CGA ACC CCG GGC GTC CGC CAT GAT ACC CTT GCG AAT TTA TCC ACC AGA CCA CCT CGA GAA CAA C-3′		
vaultRNA1-1_Fwd	5′-GTT GTT CTC GAG AGC TCA GCG GTT ACT TCG ACA GTT CTT TAA TTG AAA CAA GCA ACC TGT CTG GGT TGT TCG AGA AAG CTT GTC GTC-3′		Oligonucleotides annealed then enzyme-treated to create the adenovirus vaultRNA1-1 promoter, then inserted into pNLP3
vaultRNA1-1_Rev	5′-GAG GAC AAG CTT TCT CGA ACA ACC CAG ACA GGT TGC TTG TTT CAA TTA AAG AAC TGT CGA AGT AAC CGC TGA GCT CTC GAG AAC AAC-3′		
tRNA-Tyr_Fwd	5′-GTT GTT CTC GAG TAG CTC AGT GGT AGA GCA TTT AAC TGT AGA TCA AGA GGT CCC TGG ATC AAC TCA AGC TTG TCG TC-3′		Oligonucleotides annealed then enzyme-treated to create the human tRNA-Tyrosine(Tyr) promoter, then inserted into pNLP3
tRNA-Tyr_Rev	5′-GAC GAC AAG CTT GAG TTG ATC CAG GGA CCT CTT GAT CTA CAG TTA AAT GCT CTA CCA CTG AGC TAC TCG AGA ACA AC-3′		
TMER1-min-XhoI-F	5′-TCG AGT AGC TCA ATT GGT AGA GCA ACA GGT CAC CGA TCC TGG TGG TTC TCG GTT CAA GTC C-3′		Oligonucleotides annealed to create the TMER1-minimal promoter, then inserted into pNLP3
TMER1-min-HindIII-R	5′-AGC TTG GAC TTG AAC CGA GAA CCA CCA GGA TCG GTG ACC TGT TGC TCT ACC AAT TGA GCT A-3′		
TMER4-min-XhoI-F	5′-TCG AGT AGC TCA ATT GGT AGA GCG GCA GGC TCA TCC CCT GCA GGT TCT CGG TTC AAT CCC-3′		Oligonucleotides annealed to create the TMER4-minimal promoter, then inserted into pNLP3
TMER4-min-HindIII-R	5′-AGCTTG GGA TTG AAC CGA GAA CCT GCA GGG GAT GAG CCT GCC GCT CTA CCA ATT GAG CTA-3′		
TMER5-min-XhoI-F	5′-TCG AGT AGC TCA ATT GGT AGA GCA TCA GGC TAG TAT CCT GTC GGT TCC GGT TCA AGT CC-3′		Oligonucleotides annealed to create the TMER5-minimal promoter, then inserted into pNLP3
TMER5-min-HindIII-R	5′-AGC TTG GAC TTG AAC CGG AAC CGA CAG GAT ACT AGC CTG ATG CTC TAC CAA TTG AGC TA-3′		
Human pretRNA-Tyr-GTA-1-1_F	5′-GCCTTCGATAGCTCAGTTGGTAG-3′		To performSYBER qPCR for the pretRNA-Try-GTA-1-1 gene
Human pretRNA-Tyr-GTA-1-1_R	5′-GGATTCGAACCAGCGACCT-3′		
γHV68-TMER1-iQ_F	5′-GAGCAACAGGTCACCGATCC-3′		To performSYBER qPCR for TMER1 gene
γHV68-TMER1-iQ_R	5′-TGCAGACAAGTGATTGCACTG-3′		
NFAT5 Forward Primer	5′-CATGAGCACCAGTTCCTACAATGAT-3′		TaqMan qPCR for NFAT5 gene
NFAT5 Reverse Primer	5′-TGCTTTGGATTTCGTTTTCGTGATT-3′		
NFAT5 Probe	5′-ACGAGGTACCTCAGTGTT-3′		

a Includes sequences for primers, oligonucleotides, and probes used for cloning reporter constructs, sequencing, RT-PCR, and qPCR.

### Generating a pol III promoter-driven NanoLuc reporter panel.

All promoters were generated to include XhoI and HindIII overhang sequences on the 5′ and 3′ ends, respectively. Several promoters were constructed using ligated oligonucleotides. All sequences for primers and oligonucleotides used in this work are shown in Table 2. PCR-amplified promoters and pNLP3 were digested with XhoI and HindIII, and then promoters were ligated into pNLP3 using T4 DNA ligase (New England BioLabs, Inc.). Ligated constructs were transformed into One Shot electro- or chemically competent TOP10 Escherichia coli cells (number C404052 or number C404010; Thermo Fisher Scientific), which were then plated at several dilutions on LB agar containing ampicillin. Resulting colonies were expanded in LB broth with ampicillin, and plasmid was isolated using the QIAprep Spin miniprep kit (Qiagen). All constructs were confirmed by sequencing.

### Transfecting cells.

For transfections, HEK 293 cells were cultured in 5% FBS-DMEM without penicillin or streptomycin for approximately 24 h. Transfection solutions contained Opti-MEM (Thermo Fisher Scientific), NanoLuc plasmid (pNLP3 with inserted pol III promoters), and the Firefly control plasmid (pGL3-Control; Promega). After plasmids were added to the Opti-MEM, solutions were incubated with X-tremeGENE HP DNA transfection reagent (Sigma-Aldrich) for at least 15 min at room temperature. Transfections were performed in several plate formats, depending on the downstream use; transfection solutions were added dropwise to the appropriate wells (10 μl of solution for 96-well plates, 100 μl of solution in 12-well plates, 200 μl of solution in 6-well plates). For all transfections, the molar ratio of NanoLuc plasmid to Firefly plasmid was kept at 10:1; the total amount of DNA transfected per well was adjusted depending on the plate size (approximately 10 ng for 96-well, 100 ng for 12-well, and 200 ng for 6-well plates). Some experiments involved transfecting 2 μg of total plasmid per well in a 6-well format. Cells were incubated with transfection solution for 24 h prior to downstream applications, unless otherwise stated.

To analyze how promoter activity and pol III transcription was affected by γHV68 infection, transfected HEK 293 (for NanoLuc experiments) or 3T12 (for RNA flow) cells were infected with the WT, TMER-TKO, or EBER-KI γHV68 at a multiplicity of infection (MOI) of 1 or 5 PFU per cell. Cells were cultured for approximately 24 h prior to infection. Cell counts were determined by treating with 0.05% Trypsin-EDTA (number 25300-054; Life Tech) to remove cells. These cells were mixed with Trypan blue dye (number 145-0021; Bio-Rad) to obtain a live cell count using the TC20 automated cell counter (Bio-Rad). Virus stocks were mixed with 5% complete DMEM, added to cells, and incubated with virus for 1 h. Viral inoculum was then removed and replaced with complete 5% DMEM. For samples treated with phosphonoacetic acid (PAA; 200 μg/ml; number 284270; Sigma-Aldrich), inoculum was removed and replaced with 5% complete DMEM containing the drug. Inhibition of RNA polymerase III was achieved by treating cells with 40 μM CAS 577784-91-9 (number 557404-M; Sigma) immediately prior to transfection and again following viral inoculation.

### Dual-luciferase assays.

Following transfection and infection, HEK 293 cell lysates were collected for analysis of luciferase activity. All luciferase assays were performed using the Nano-Glo dual-luciferase reporter assay system (Promega). For experiments performed in the 12-well format, supernatant was removed and cells were scraped, collected into 1.5-ml tubes, and then pelleted. Cell pellets were resuspended in 250 μl of passive lysis buffer per the manufacturer’s protocol. Eighty microliters of the cell suspensions were used for assays. For luciferase assays performed in the 96-well format, 80 μl of ONE-Glo EX reagent was added directly to the cells and supernatant, as recommended by the manufacturer. Samples were incubated at room temperature while on a shaker for 3 min. This solution was then transferred from a transparent 96-well culture plate to a white-wall luminescence plate prior to reading Firefly luciferase activity on the Tecan Infinite 200 PRO plate reader. Next, 80 μl of the NanoDLR Stop & Glo reagent was added to the solution and incubated at room temperature on a shaker for at least 10 min. Samples were read on the Infinite 200 PRO again for the NanoLuc luminescence measurement.

### RT-PCR and RT-qPCR.

RNA was isolated from transfected HEK 293 cells with TRIzol reagent (number 15596026; Thermo Fisher Scientific) and DNase treated with TURBO DNase (number AM2238; Invitrogen) by following the manufacturer’s protocols. RNA amplification and removal of DNA was confirmed by RT-PCR or PCR amplification of the NanoLuc gene and a control host gene, 18S. RT-PCR was performed using the OneStep RT-PCR kit (number 210212; Qiagen), with the following conditions: (i) 50°C for 30 min, (ii) 95°C for 15 min, (iii) 40 cycles of 94°C for 30 s, 52°C for 30 s, and 72°C for 30 s, (iv) 72°C for 10 min, and (v) hold at 4°C. PCR was performed using *Taq* DNA polymerase (number 201205; Qiagen) with the following conditions: (i) 95°C for 5 min, (ii) 40 cycles of 94°C for 30 s, 52°C for 30 s, and 72°C for 30 s, (iii) 72°C for 10 min, and (iv) hold at 4°C. RNA samples that showed no product following PCR amplification were deemed DNA-free and converted to cDNA using SuperScript III reverse transcriptase (number 18080093; Invitrogen) by following the manufacturer’s protocol. One hundred nanograms of the cDNA was then used for qPCR analysis of the NanoLuc, human pretRNA-Tyr-GTA-1-1, γHV68 TMER1, and human 18S genes using the iQ SYBR green supermix (number 1708880; Bio-Rad) with the following conditions: (i) 95°C for 3 min, (ii) 40 cycles of 95°C for 15 s, 60°C or 61°C for 1 min, and (iii) 95°C for 15 s, 60°C for 1 min, and 95°C for 15 s. Amplification of NanoLuc, pretRNA, or TMER1 was normalized to 18S expression to calculate the relative difference of target gene expression using the Pfaffl method: $\frac{{\text{NanoLuc primer efficiency}}^{\text{Target}\;\Delta \mathrm{Ct}}}{18{\text{S primer efficiency}}^{18S\;\Delta \mathrm{Ct}}}$ (53). A single product for each target was confirmed by melt curves and gel electrophoresis of product following qPCR.

NFAT5 expression was measured using primer-probe qPCR of 100 ng cDNA with the VIC probe and black hole quencher (BHQ). Conditions for the qPCR were (i) 95°C for 10 min, (ii) 40 cycles of 95°C for 10 s, 60°C for 30 s, and 72°C for 1 s, and (iii) 40°C for 30 s. Quantity of NFAT5 was determined using a plasmid-based standard curve.

### PrimeFlow RNA assay.

The PrimeFlow RNA assay kit (number 88-18005-210; Thermo Fisher Scientific) was used to analyze expression of noncoding RNAs in mock- and γHV68-infected 3T12 cells. Cells were infected at an MOI of 1 or 5 for 16 h and then processed by following the manufacturer’s protocol. Probes used were TMERs (type 4/AF488 or type 1/AF647), EBERs (type 1/AF647), ORF18 (type 4/AF488), and U6 snRNA or 4.5 rRNA (type 6/AF750), with compatible probe labels depending on the experiment. Samples were collected on an LSR II flow cytometer (BD Biosciences) and included single-stain and “full minus one” controls. Flow cytometry data were analyzed using FlowJo software (version 10.6.1), with compensation based on single stained beads and cells. Compensated flow cytometry data were subsequently analyzed for singlet events based on doublet discrimination, as exemplified in Fig. 7. Distinctions of negative and positive populations were based on control samples, as shown in Fig. 8 and 9.

### Software and statistical analysis.

Statistical analysis and graphing were done in GraphPad Prism (version 8.0d). Statistical significance was tested by unpaired *t* test (comparing two conditions), one-way analysis of variance (ANOVA) (comparing three or more conditions), or two-way ANOVA (comparing grouped data) and subjected to multiple-correction tests using recommended settings in Prism. All flow cytometry data were analyzed in FlowJo (version 10.6.1) with flow cytometry data shown as pseudocolor dot plots.
